# Polybrominated diphenyl ether serum concentrations in a Californian population of children, their parents, and older adults: an exposure assessment study

**DOI:** 10.1186/s12940-015-0002-2

**Published:** 2015-03-14

**Authors:** Xiangmei (May) Wu, Deborah H Bennett, Rebecca E Moran, Andreas Sjödin, Richard S Jones, Daniel J Tancredi, Nicolle S Tulve, Matthew Scott Clifton, Maribel Colón, Walter Weathers, Irva Hertz-Picciotto

**Affiliations:** Department of Public Health Sciences, University of California, One Shields Avenue, MS1C, 95616 Davis, CA USA; Centers for Disease Control and Prevention, National Center for Environment Health, Atlanta, GA USA; Department of Pediatrics, University of California, Davis, CA USA; U.S. Environmental Protection Agency, Office of Research and Development, National Exposure Research Laboratory, Human Exposure and Atmospheric Sciences Division, Research Triangle Park, NC USA

**Keywords:** Children, Flame retardants, PBDEs, Serum concentration, Temporal variability

## Abstract

**Background:**

Polybrominated diphenyl ethers (PBDEs) are used as flame retardants in many household items. Given concerns over their potential adverse health effects, we identified predictors and evaluated temporal changes of PBDE serum concentrations.

**Methods:**

PBDE serum concentrations were measured in young children (2-8 years old; N = 67), parents of young children (<55 years old; N = 90), and older adults (≥55 years old; N = 59) in California, with concurrent floor wipe samples collected in participants’ homes in 2008-2009. We also measured serum concentrations one year later in a subset of children (N = 19) and parents (N = 42).

**Results:**

PBDE serum concentrations in children were significantly higher than in adults. Floor wipe concentration is a significant predictor of serum BDE-47, 99, 100 and 154. Positive associations were observed between the intake frequency of canned meat and serum concentrations of BDE-47, 99 and 154, between canned meat entrees and BDE-154 and 209, as well as between tuna and white fish and BDE-153. The model with the floor wipe concentration and food intake frequencies explained up to 40% of the mean square prediction error of some congeners. Lower home values and renting (vs. owning) a home were associated with higher serum concentrations of BDE-47, 99 and 100. Serum concentrations measured one year apart were strongly correlated as expected (r = 0.70-0.97) with a slight decreasing trend.

**Conclusions:**

Floor wipe concentration, food intake frequency, and housing characteristics can explain 12-40% of the prediction error of PBDE serum concentrations. Decreasing temporal trends should be considered when characterizing long-term exposure.

**Electronic supplementary material:**

The online version of this article (doi:10.1186/s12940-015-0002-2) contains supplementary material, which is available to authorized users.

## Background

Polybrominated diphenyl ethers (PBDEs) were used as flame retardants in furniture, electronics, as well as many baby products. Many potential adverse health effects have been reported due to exposure to PBDEs, i.e., endocrine disruption, reproductive and developmental toxicity, central nervous system effects, and immune system impact [1-3]. PBDEs do not easily degrade in the environment and many of the congeners have relatively long half-lives in the body [4]. Reported levels of PBDEs found in the human body increased between the 1980s and early 2000s in the U.S. [5].

California had stringent flammability standards (TB117) enacted in the 1970s, and thus manufacturers added flame retardants into a wide variety of furniture and baby products [6]. PBDEs were phased out of the European market by 1999. Though the major U.S. manufacturers had voluntarily stopped producing and importing PentaBDE and OctaBDE in 2004, the existing stock of these PBDEs continued to be used and large quantities remained in indoor environments in products in use. In 2006, the manufacture, distribution and processing of products containing these two commercial mixtures of PBDEs were banned in California, 1-2 years earlier than other parts of the U.S. Studies have reported higher house dust and serum PBDE levels in California than other U.S. states and European countries [7,8]. Therefore, it is necessary to continue to measure PBDE exposure after the manufacturing changes, especially among the sensitive group of young children.

Further, PBDE concentrations are commonly measured in human serum, which can be difficult to obtain, especially from infants and young children. Using information such as environmental measurements, dietary habits, or other family members’ serum concentrations to categorize exposure could be an alternative in situations where blood cannot be obtained. Previous studies using exposure models coupled with measured data have indicated that ingestion of dust is an important contributor of exposure in the U.S. [9]. Food intake is the other main contributor to exposure, with ingestion of fish, meat and dairy as the major sources of PBDEs [10-12]. Further information is needed on the relative contribution of these exposure routes.

Therefore, we conducted a series of analyses to explore the possibility of predicting body burdens of PBDEs, to understand whether we can quantify PBDE serum concentrations using measured environmental concentration, housing characteristics, or food intake frequency. Also, we explored the possibility of estimating one’s serum PBDE concentration using the available measurement of a family member. This would be especially important for estimating children’s exposure, as it is usually more difficult to collect blood samples from children. Lastly, to determine the exposure time window represented by a single sample, it is necessary to learn how PBDE serum concentrations change over time. This study provides valuable data of repeat measurements one year apart from the same individuals to characterize temporal variation of PBDE exposure.

## Methods

### Study population

Participants were recruited by contacting households enrolled in the Study of Use of Products and Exposure-Related Behavior (SUPERB), which collected information on consumer product use, such as pesticides, cleaning products, and personal care products, food intake frequency, and time-activity patterns. SUPERB included two sub-populations: 499 homes with young children born between 2000-2005 that were randomly identified through birth certificate records from selected northern California counties, and 156 households with older adults that were selected randomly from housing units in the southern portion of California’s Central Valley. More details on the SUPERB study design and recruitment can be found in Hertz-Picciotto et al. [13].

**Figure 1:**
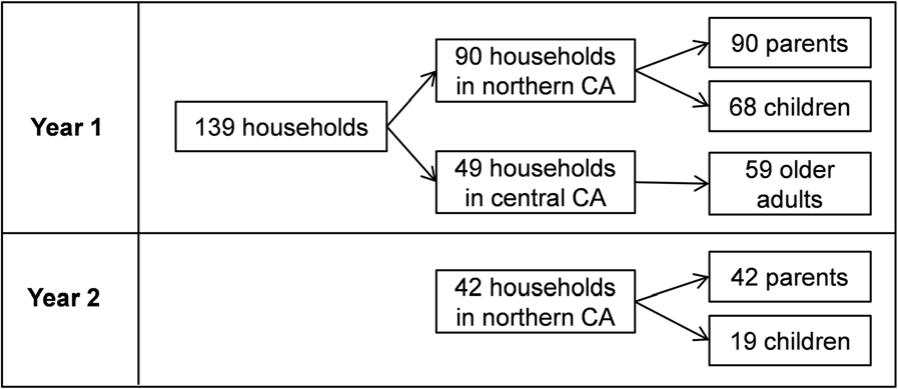
**Study population illustration chart.**

As an add-on to SUPERB, between 2008-2009, a total of 139 households participated in this study that involved visits to the homes and collection of bio-specimens and environmental samples (Figure 1). This includes 90 households with an adult parent (referred to as “parent” below) and a young child under the age of 8 years (93% of children were 5 years and under), and 49 households with an older adult participant (96% ≥55 years). Forty-two households with a parent-child pair were visited a second time approximately one year (mean = 341 days, range 225-505 days) after their first study visit to estimate temporal variability of serum and environmental measures.

All recruitment and data collection protocols were approved by the Institutional Review Board at the University of California, Davis, and the Centers for Disease Control and Prevention (CDC), and informed consent for participation was obtained upon enrollment into the study.

### Sample collection

Trained field staff visited participants’ homes to collect floor wipe samples and blood samples from adult and child participants. In addition, blood samples were collected from ten spouses of participating older adults. Blood samples were drawn into 10-mL red top Vacutainer™ tubes (Becton-Dickinson, Rutherford, NJ) with no anticoagulant by a trained phlebotomist and then transported on ice. Blood samples were allowed to clot for at least two hours and were centrifuged at 2400 rpm for 15 minutes. To obtain maximum serum yield, some samples were centrifuged a second time for 10 minutes. A 4-6 mL aliquot of the serum was transferred into a pre-cleaned 10-mL brown glass bottle. All processing was conducted in a laboratory fume hood to limit introduction of dust particles. Aliquots were stored at -80 ºC until shipped on dry ice to CDC (Atlanta, GA) for analysis.

A floor wipe sample was also collected from the kitchen (approximately 1.5 m^2^ section) in each participant’s home, using cotton Twillwipes (M.G. Chemicals, P/N 829-50). Samples were collected in the kitchen as this is often the only room, other than bathrooms without carpeting. The Twillwipes were pre-cleaned and dampened with 4.0 mL of pesticide residue analysis grade isopropanol before use. Details on the sample collection have been reported [14].

Field staff completed a walk-through questionnaire at the home visit. During the walk-through, participants were asked the age and size of their home and whether they rented or owned. Field staff recorded the type of home and counted the number of pieces of upholstered or foam furniture items in room(s) where dust and air samples were collected, with participants providing the age of these pieces of furniture. We also collected information about age, size and use of televisions (TVs).

A phone interview was conducted following the home visit, collecting housing characteristics and a food frequency questionnaire for food items commonly consumed by Americans and hypothesized to contain high level of PBDEs.

### Sample analysis

BDE-17, 28, 47, 66, 85, 99, 100, 153, 154, 183, and 209 were measured. Polybrominated biphenyl 153 (BB-153), which was used as a flame retardant for many years but was phased out in 1976, was also measured. The sample extraction and lipid removal procedure has been published [15]. Briefly, serum samples were extracted using an automated liquid/liquid extraction followed by lipid removal using a two-layered silica/silica-sulfuric acid column. Final determination of target analytes was performed by isotope dilution gas chromatography high-resolution mass spectrometry. The limit of detection (LOD) varies by the available sample volume for each sample, with ranges given in Additional file 1: Table S1. Detailed analytical and QA/QC procedures for environmental and serum samples have been presented by Clifton et al. [14] and Sjodin et al. [15], respectively. The resulting distributions from these homes, along with QA/QC data, have also been reported previously [16].

### Data analysis

Serum concentrations were lipid adjusted. Summary statistics for the three age classes: young children, parents, and older adults were calculated from the first-year data. Detection rates varied by congener. The SAS procedure, PROC LIEFREG, was used to calculate geometric means (GM), using maximum likelihood estimation (MLE) methods for lognormal distributions subject to left-censoring at the LOD [17], and to compare GMs across age classes. Comparisons were restricted to congeners with detectable serum levels in at least 60% of samples.

Further correlation, mean comparisons and mixed-effects linear regression analyses were restricted to congeners with high detection rates (over 80%), replacing serum concentration values below the LOD with LOD divided by the square root of 2 and then log-transforming serum concentrations. Spearman correlation coefficients were calculated between baseline PBDE serum concentrations of the parent and child from the same household as well as between serum levels in older participants and their spouse, respectively.

Serum concentrations for six major PBDE congeners, BDE-47, 99, 100, 153, 154 and 209, were evaluated in mixed-effects multiple regression models to identify potential predictors. Because the dependent variable is log-transformed, regression coefficients are sometimes back-transformed via exponentiation and reported as adjusted geometric mean ratios. Due to the low detection of BDE-154 and 209, a logistic regression model was used to evaluate predictors of the detection (yes/no). Three rounds of modeling were conducted: 1) Regression with floor wipe PBDE concentrations. The floor wipe is being considered here as a proxy for exposures associated with the indoor residential environment [16]. 2) Regression with floor wipe concentrations and food intake frequency to examine if food intake adds extra power to the prediction of PBDE concentrations. 3) Regression with housing characteristics, to explore the possibility of replacing environmental measurement with housing characteristics collected by questionnaires or from public sources, which cost less to obtain. A random effect was specified in the mixed-effects model to account for within-household correlation, considering that parents and children or the older couples from the same household may share similarities in regard to diet and housing condition.

In multilevel models and in logistic regression models, the coefficient of determination (R-square) is not uniquely defined. For the mixed-effects linear regression model, we report restricted maximum likelihood estimates of within-household (Level 1) and between-household (Level 2) variance components for the full model (all predictors plus random household intercepts) as well as for a null model (random household intercepts only). For a given model, the ratio of the between-household variance component to the sum of the between- and within-household variance component estimates the intra-cluster (within-household) correlation coefficient, a measure of the correlation in the residuals from observations from the same household. Singer and Willett define pseudo R-squares for each variance component as the proportion reduction in the component in comparing the larger model to the smaller model [18]. The pseudo R-square at the between-household level quantified the impact of the between-household covariates, especially the dust-wipe predictor. Snijders and Bosker define a pseudo R-square for the level 1 observations as the proportional reduction in level 1 mean square prediction error (the sum of the variance components) [19]. For logistic regression, we report Nagelkerke’s proposed coefficient of determination [20].

Food items evaluated include dairy products (i.e., milk, cheese, yogurt, ice cream, butter), meats (i.e., pork, beef, poultry, canned meat, canned meat entrées), fish (including salmon, tuna, white fish, and other freshwater fish), and fast food. Food intake frequency was recorded in categorical format, including choices of “Never”, “Less than once per week”, “Once per week”, “2-3 times per week”, “4-6 times per week”, “Once per day”, and “More than once per day”, and then converted into number of times per week (0, 0.5, 1, 2.5, 5, 7, 14, respectively). Portion sizes were not recorded.

Housing variables included dwelling type (apartment vs. single family home), ownership (rent vs. own), age, value, size, and the number of pieces of furniture in the sampled room. The age and size were confirmed using publicly available data. House age was categorized as built before or after 1977 when the implementation of California Title 24 led to more airtight houses [21]. Home value was the estimated market value as determined by a web-based service (www.Zillow.com) in December 2008. We note that in California, home values are not reassessed by counties at a rate greater than 2% per year; therefore, accurate estimates are not available from county assessor records.

In addition, we tested if adults’ education level, a more common socioeconomic indicator, can replace home value in predicting PBDE concentrations. Adults’ education level was categorized into “no college”, “some or complete college”, and “postgraduate”.

Temporal variability was examined among households visited twice, including 42 parents and 19 young children. Both Spearman correlation coefficients and the percent of change were calculated. The serum level change between the two visits was tested against zero using the paired T-test (for normally distributed congeners, visually determined based on distribution graph) or the paired sign test (for non-normally distributed congeners). All statistical analyses were conducted using SAS 9.2 for Windows® (SAS Institute Inc., Cary, NC, USA). Statistical significance was set as α = 0.05 (two-sided).

## Results

### Demographics

Population demographics are presented in the Supplemental Materials (Additional file 2: Table S2). A total of 68 young children, 90 parents, and 59 older adults provided serum. Note that the majority (83%) of the parents were female. In addition, though we over-sampled the households in which the mother had lower education attainment, our study population had a higher education attainment, 99% having a high school diploma compared to 80.8% of California population [22], indicating a higher socioeconomic status.

### Distribution of PBDE congener serum concentrations

Summary statistics of PBDE serum concentrations for the three age classes are presented in Table 1. BDE-47 had the highest GM serum concentration in all three age classes, followed by BDE-153, 99 and 100. BDE-17, 66, 183 and 209 were detected in fewer than 25% of samples. Geometric means should be interpreted with caution for congeners with low detection frequencies Correlations between congeners were similar to those found in other studies and are presented in the Supplemental Materials (Additional file 3: Table S3).

**Table 1:** **Distribution of PBDE serum concentrations (ng/g lipid) in three age classes in California at baseline visit** ^**a**^

**PBDE**	**Overall % detected**	**Children (N = 68)**				**Parents of young children (N = 90)**				**Older adults (N = 59)**				**Diff by age (** ***p*** **-value)** ^**b**^
		**% detected**	**GM**	**P75**	**P95**	**% detected**	**GM**	**P75**	**P95**	**% detected**	**GM**	**P75**	**P95**	
BB-153	80%	60%	0.92	1.80	5.40	82%	1.22	2.10	5.00	100%	2.54	3.40	8.90	<0.001
BDE-17	18%	40%	0.34	0.85	1.70	7%	0.05	—	0.70	10%	0.08	—	2.00	—
BDE-28	84%	84%	2.25	4.55	11.6	93%	1.41	2.50	5.20	71%	1.30	3.10	12.3	0.004
BDE-47	100%	100%	61.8	115	364	100%	20.8	39.8	94.2	100%	25.4	44.3	193	<0.0001
BDE-66	22%	38%	0.41	0.80	1.50	12%	0.14	—	0.70	19%	0.19	—	1.30	—
BDE-85	53%	76%	1.29	2.55	6.60	44%	0.36	0.80	2.20	41%	0.38	0.80	4.90	—
BDE-99	99%	99%	13.0	24.2	77.9	98%	3.78	6.30	26.1	100%	4.11	9.30	46.2	<0.0001
BDE-100	99%	100%	12.7	21.1	64.0	98%	3.77	7.30	21.0	100%	4.40	6.50	60.0	<0.0001
BDE-153	100%	100%	16.1	26.2	86.7	100%	6.76	15.0	48.9	100%	7.32	11.9	101	<0.0001
BDE-154	51%	74%	1.11	2.20	5.30	38%	0.31	0.60	1.80	46%	0.40	0.80	4.30	—
BDE-183	15%	15%	0.20	—	0.80	12%	0.13	—	0.50	19%	0.18	—	0.80	—
BDE-209	16%	24%	2.76	—	9.60	8%	1.25	—	8.00	20%	1.76	—	7.40	—

^a^Analyses include values below the limit of detection. Geometric means (GM) are maximum likelihood estimates for log-normally distributed data left-censored at the LOD. GM for values with low detection percentages should be interpreted with caution.

^b^Pr > |Wald Chi-square| to test the null hypothesis that geometric mean PBDE serum concentrations are equal for the three groups. Comparisons performed only when at least 60% of values above detection limits in all three groups.

Statistical significance testing of geometric mean baseline serum level comparisons were reported for congeners with greater than 60% detection frequency in each age group. Children generally had higher serum concentrations of PBDEs than parents and older adults, with significantly higher concentrations for major pentaBDE congeners (Table 1). In paired t-tests, we found that children had significantly higher concentrations (p < 0.01) than their own parents for BDE-28, 47, 99, 100 and 153, but not for BB-153. For BB-153, older adults had the highest serum concentrations.

Baseline PBDE serum concentrations of parents and children from the same household were significantly correlated for major congeners listed in Table 2, with Spearman correlation coefficients ranging between 0.66 and 0.74. In tertile analysis using age-specific percentiles to classify serum levels into low, middle and high groups, weighted Cohen’s kappa values ranged from 0.38 to 0.57. In contrast, within the older couples, significant correlations were only observed for BDE-28, 47, 99 and 153, but not for BB-153 and BDE-100.

**Table 2:** **Correlation between the PBDE serum concentrations of family members at the baseline visit** ^**a**^

	**Child vs. Parent (N = 68)**	**Within older couple (N = 10)**
BB-153	0.42 [0.19, 0.59]*	0.05 [-0.60, 0.66]
BDE-28	0.67 [0.51, 0.78]*	0.78 [0.27, 0.94]*
BDE-47	0.74 [0.60, 0.83]*	0.72 [0.13, 0.92] *
BDE-99	0.70 [0.56, 0.81]*	0.74 [0.17, 0.93]*
BDE-100	0.66 [0.50, 0.78]*	0.45 [-0.27, 0.83]
BDE-153	0.68 [0.53, 0.79]*	0.72 [0.13, 0.92]*

^a^Expressed as Spearman Correlation Coefficients and 95% confidence interval (based on Fisher Z transformation) in brackets. Concentrations below LODs replaced by LOD/√2. Correlation was not calculated for BDE-17, 66, 85, 154, 183, and 209 due to low percentage of detects.

^*^ Statistically significant at α = 0.05 (two-sided).

### Factors predicting PBDE serum concentrations

Predictor analysis starts with a regression model with age class and floor wipe concentrations of PBDEs (Additional file 4: Table S4). All age classes were included. Age class was a significant predictor of PBDE serum concentrations, with young children having higher serum concentrations of pentaBDEs than parents and older adults. Both young children and older adults had higher BDE-209 serum concentrations than parents of young children. The variation was consistent with the differences among the three age classes discussed earlier. Take BDE-47 as an example, the reference is the group of parents of young children, the regression coefficient for the older adult group (β = 0.27, p = 0.12) suggests their geometric mean concentrations were 31% (=e^0.27^-1) higher than the reference group, though not statistically significant. The young children group has 210% higher (β = 1.14, p < 0.01) geometric mean serum concentrations than the reference group. Floor wipe concentration is a significant predictor of serum levels of BDE-47, 99, 100 and 154 (*p* < 0.01). The linear regression coefficients for floor wipe concentrations ranged from 0.03 to 0.15 for BDE-47, 99, and 100 in mixed effect models of log-transformed serum concentrations, which means for every 1 pg/cm^2^ increase of the floor wipe concentration, the geometric mean serum concentration would increase by a factor ranging from 1.03 to 1.16. The logistic regression coefficient for the floor wipe concentration was 0.82 for the detectable BDE-154 dependent variable, indicating that the odds of detecting BDE-154 in the blood increased by a factor of 2.27 for every 1 pg/cm^2^ increase of the BDE-154 floor wipe concentration. Based on the R-square values, age class and floor wipe concentrations explain between 10-30% of the mean square prediction error of the serum concentrations, depending on the congener.

When adding food intake frequency in the model, the age class effect remained the same for the pentaBDE congeners we examined (Table 3). For BDE-209, the regression coefficients suggested that the older adult group had the highest concentrations. Young children’s levels were higher than their parents’, but not statistically significant. Floor wipe concentration remained a significant predictor for serum levels of BDE-47, 99, 100 and 154, and it became a significant predictor for BDE-209. The regression coefficients for floor wipe concentrations were similar to those of the models with age group and floor wipe concentration only. BDE-153 serum concentrations were not associated with the floor wipe concentrations in either model. Among the twelve types of food we tested, canned meat is positively associated with serum levels of BDE-47, 99, 100 and 154 (*p* = 0.01-0.06), with linear regression coefficients ranging from 0.55 to 0.89 and logistic regression coefficient being 1.67 for BDE-154. In other words, the geometric mean serum concentration of BDE-47, 99 and 100 would increase by a factor ranging from 1.73 to 2.44 and the odds of detecting BDE-154 in the blood increased by a factor of 5.31 for having canned meat one more time per week. Increasing the frequency of having canned meat entrée by one more time per week could increase the odds of detecting BDE-154 and 209 in the blood by a factor of 2.55 and 3.48, respectively. Having tuna and white fish one more time per week would increase the geometric mean serum concentration of BDE-153 by a factor of 1.43 (*p* = 0.02). Having pre-packaged candy or bakery items occasionally (less than once per week) could increase the odds of detecting BDE-209 in the blood by a factor of 6.92, compared to those never having such food. Unexpected, pork is negatively associated with serum BDE-154 (*p* = 0.02) and we cannot speculate on the reason for this. No significant association with PBDE serum concentrations was observed for beef, poultry, dairy fat, salmon, freshwater fish, canned fish, or fast food.

**Table 3:** **Results of multiple regression models predicting log-transformed PBDE serum concentrations (ng/g lipid)** ^**a**^

**Effects**	**BDE-47**	**BDE-99**	**BDE-100**	**BDE-153**	**BDE-154**	**BDE-209**
N	162	159	160	162	162	158
Intercept	2.67(0.27)**	0.83(0.29)	0.95(0.27)	1.72(0.33)	-2.28(0.79)	-2.44(1.14)
Age class (ref = parents of young children)						
Young children	1.24(0.19)**	1.40(0.21)**	1.38(0.19)**	1.18(0.23)**	2.41(0.57)**	1.16(0.82)
Older adults	0.32(0.20)	0.11(0.22)	0.24(0.21)	0.21(0.25)	0.47(0.52)	2.31(0.90)*
Floor wipe concentration (pg/cm^2^)	0.03(0.01)**	0.03(0.01)*	0.12(0.03)**	-0.01(0.02)	0.69(0.25)**	0.01(0.003)*
Frequency of having fast food (ref = never)						
<1 time/week	-0.18(0.24)	-0.02(0.26)	-0.06(0.24)	-0.10(0.29)	1.15(0.66)	-1.05(0.89)
≥1 time/week	-0.09(0.24)	-0.04(0.26)	0.12(0.24)	-0.04(0.29)	1.01(0.64)	-1.04(0.92)
Frequency of having pre-packed candy or bakery (ref = never)						
<1 time/week	0.37(0.21)	0.35(0.23)	0.51(0.22)^+^	0.42(0.25)	0.59(0.54)	1.93(0.88)*
≥1 time/week	0.05(0.19)	-0.05(0.21)	0.005(0.19)	0.001(0.23)	-0.32(0.51)	0.36(0.81)
Frequency of eating (time/week)						
dairy (based on dairy fat intake)	-0.02(0.01)	-0.002(0.01)	-0.01(0.01)	-0.01(0.01)	-0.03(0.03)	-0.02(0.04)
Beef	-0.03(0.05)	-0.06(0.05)	-0.03(0.05)	-0.07(0.06)	0.04(0.13)	0.21(0.19)
Pork	-0.05(0.06)	-0.08(0.07)	-0.09(0.06)	-0.08(0.07)	-0.46(0.20)*	-0.23(0.36)
Poultry	0.04(0.04)	0.04(0.04)	-0.002(0.04)	0.001(0.05)	0.05(0.10)	-0.09(0.17)
canned meat	0.67(0.26)*	0.89(0.27)*	0.55(0.26)^+^	0.14(0.31)	1.67(0.77)*	-10.05(6.47)
canned meat entrées	-0.04(0.10)	-0.05(0.11)	0.03(0.10)	-0.03(0.12)	0.94(0.38)*	1.25(0.50)*
canned fish	0.06(0.13)	-0.002(0.14)	0.06(0.13)	0.19(0.15)	-0.57(0.42)	-0.34(0.60)
fresh tuna and white ocean fish	0.12(0.10)	0.07(0.11)	0.17(0.10)	0.36(0.12)*	0.33(0.26)	0.68(0.40)
Salmon	0.05(0.15)	0.20(0.16)	0.06(0.15)	-0.06(0.18)	0.51(0.39)	-0.38(0.63)
fresh water fish	-0.13(0.18)	-0.08(0.20)	-0.13(0.18)	-0.03(0.22)	-0.58(0.50)	-1.73(1.34)
Variance components^b^: Full Model (Null Model)						
Between-household (Level 2)	0.64 (0.92)	0.51 (0.93)	0.59 (0.97)	0.84 (1.07)		
Within-household (Level 1)	0.15 (0.22)	0.42 (0.42)	0.24 (0.24)	0.34 (0.26)		
Intra-cluster correlation coefficient	0.81 (0.81)	0.55 (0.69)	0.71 (0.80)	0.71 (0.80)		
Singer & Willett Pseudo R-square^c^						
Household variance component	0.30	0.45	0.38	0.21		
Residual error variance component	0.30	0.00	-0.01	-0.29		
Pseudo R-square	0.30^d^	0.31^d^	0.31^d^	0.12^d^	0.38^e^	0.40^e^

^a^BDE-47, 99, 100, 153 were analyzed by generalized linear mixed-effect model, and BDE-154 and 209 were analyzed by logistic regression model due to their low detection. Results in top section of table are estimated regression coefficients (and standard error).

^b^Variance components estimated by restricted maximum likelihood estimates and reported for full model as well as for the so-called null model that contains only random household intercepts and no fixed effects. Intra-cluster correlation coefficient is the ratio of the between-household variance component to the sum of the between- and within-household variance components.

^c^Singer & Willett Pseudo R-squares describe for each variance component the proportional reduction in the null model variance component attributed to the full model [18].

^d^R-square for mixed-effects linear regression model are reported as the proportional reduction in mean squared prediction error, using Snijder and Bosker’s estimator for a two-level random-intercepts model (the proportional reduction in the sum of variance components) [19].

^e^R-square of the logistic regression model is the max-scaled R-square [20].

^**^p-value < 0.01; ^*^p-value < 0.05; ^+^marginally statistically significant, p = 0.05-0.07

When floor wipe concentrations were replaced by housing variables in the regression analysis, results suggested that home value, dwelling type, and ownership were significant predictors for various congeners (Additional file 5: Table S5). Higher home value was associated with lower serum concentrations of BDE-47, 99 and 100 (β = -0.68 to -0.63, *p* < 0.05), with the geometric mean serum concentration decreasing by a factor of 0.51 to 0.53 for every one million dollar increase of the house value. Because home value is a socioeconomic indicator, we then replaced it with a more common socioeconomic indicator, education level. Education level did not have any significant association with serum concentrations. Home value may be an alternative measure of socioeconomic status that can be utilized by future studies. The serum concentrations of BDE-153 were 66% higher among renters than owners (*p* = 0.048), and 46% higher among single family home dwellers than apartment dwellers (*p* = 0.029). No housing variables were significantly associated with detection of BDE-154 and 209 in serum. However, when employing the stepwise selection option, homes built after 1977 had a higher detection frequency of serum BDE-209 (*p* = 0.046). Other housing variables such as size, age, and window open frequency were not statistically associated with serum levels. The number of upholstered furniture items purchased between 1980 and 2004 in the sampled room and the age of the mattress were not associated with serum pentaBDEs. TV size, age, and hours of watching TV were evaluated for serum BDE-209 but not found significant, either.

The model with floor wipe concentrations explains 10-30% of the mean square prediction error of the serum PBDE concentrations. By adding food intake frequency variables, the total variance explained increased to 12-40% (Table 3). Specifically, for BDE-47, the model with food frequency reduced the within-household variance component by 43% but increased between-household variance component by 20%, namely, adding the food intake frequencies to the model explains a greater portion of the difference between households but reduced the difference between the individuals from the same household. The prediction for other congeners is also improved but less significantly than for BDE-47. Only 12-22% of total prediction error was explained by the model with housing characteristics. Based on these results, environmental concentrations combined with food intake frequency could explain a moderate portion of exposures measured by biologic concentrations of PBDEs in serum.

### Temporal variability

As expected, serum concentrations between the two visits one year apart were highly correlated for PBDE congeners with high detection rates, including BDE-28, 47, 99, 100 and 153, with Spearman correlation coefficients of 0.82-0.92 for the young children and 0.84-0.97 for the parents, indicating samples from a single time point can represent a longer time window of exposure.

**Figure 2:**
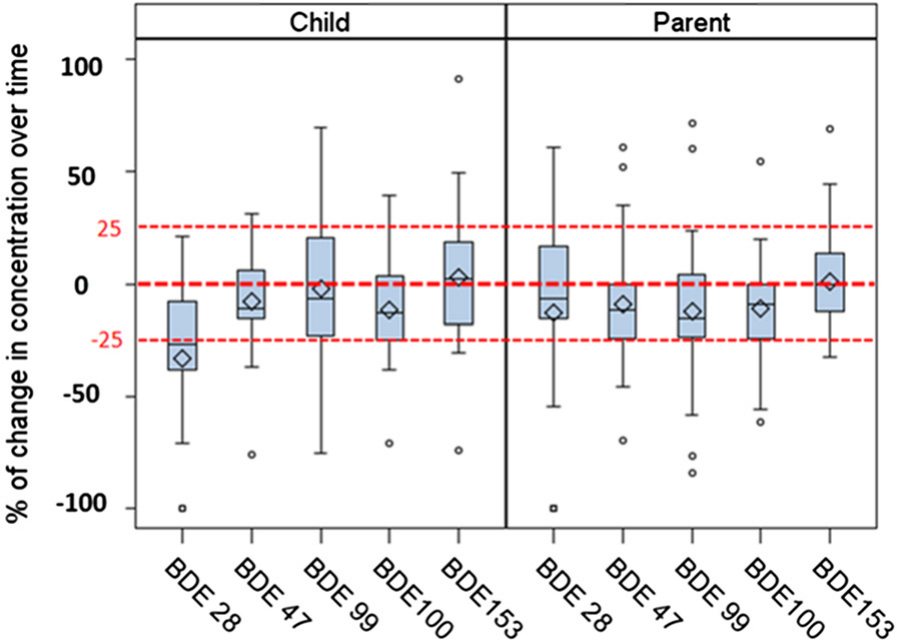
**Percent of change of selected PBDE congeners between two visits one-year apart.**

The distributions of the percent changes in serum concentrations in the one-year interval indicate concentrations were fairly consistent with a slight decreasing trend for all congeners except BDE-153. Depending on congeners, 47-78% of children had similar serum concentrations with a change < ±25% from one year to the next (Figure 2). Greater decreasing trends were observed on children’s serum concentrations of BDE-28 and 100, with a statistically significant decrease of BDE-28 in children (p < 0.01). Parents’ serum PBDE concentrations were more consistent over time than children’s, with the majority of parents (64-85% depending on congeners) having similar serum concentrations (<±25% of change) from one year to the next. About 20% of parents had >25% decrease in BDE-28, 47, 99 and 100 concentrations, however, only BDE-100 had a statistically significant decrease (p = 0.01). For the rest of the congeners we tested, the two observations one year apart were not significantly different (p > 0.05).

## Discussion

This study was designed to better understand serum PBDE concentrations for three age classes of population in California, including a focus on young children, and to explore potential predictors of PBDE serum concentrations. The results obtained from this study provide updated data on PBDE exposures in California, and provide support for potentially reducing the number of needed measurements in future epidemiology studies.

**Figure 3:**
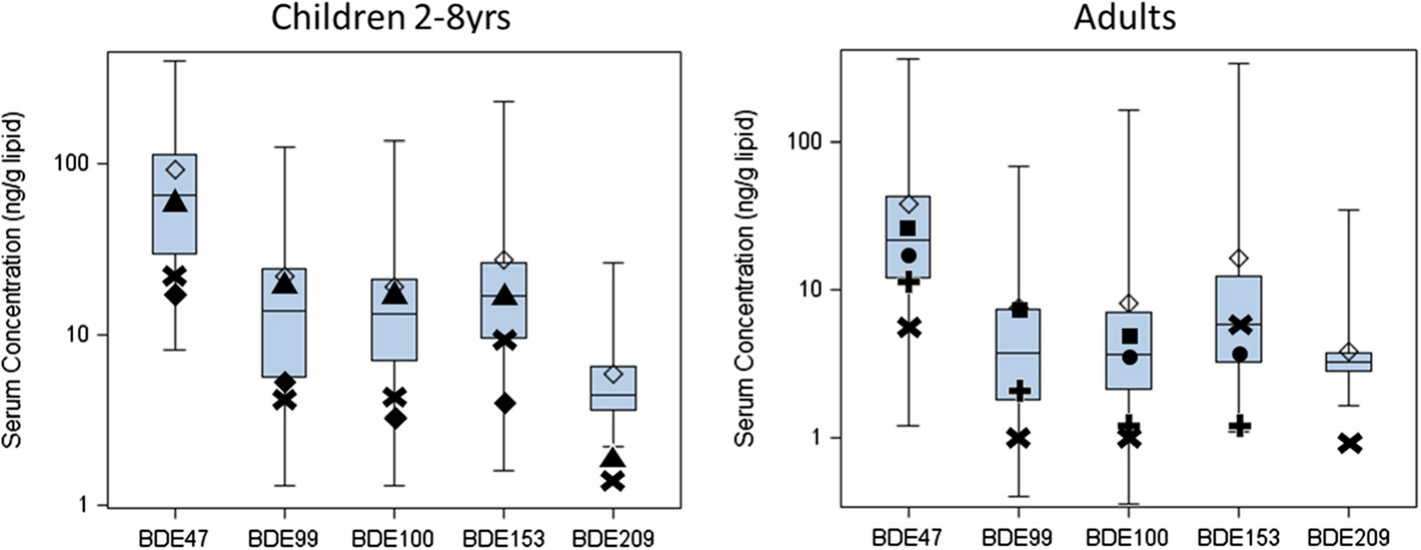
**Comparison between literature data with serum PBDE concentrations observed in this study.** Our observations are shown in the boxes, with medians labeled by the line in the middle of the box and arithmetic means labeled by the diamond. Data from other studies are labeled as: ▲ CA Children (2-5 yrs), median (Rose et al. [7]); ✖ U.S. Children (1.5-4 yrs) and Mothers, median (Lunder et al. [23]); ■ North Carolina Children (12-36 months), GM (Stapleton et al. [24]); ■ CA Adults, NHANES, GM (Zota et al. [8]); ● Non-CA U.S. Adults, NHANES, GM (Zota et al. [8]); ✚ CA Adults (mostly non-US born), median (Castorina et al. [25]).

The children's PBDE serum concentrations we observed were in the same range as those reported for California children between 2003-2005 by Rose et al. [7], as seen in Figure 3 (BDE-209 was not be compared). However, our mean levels were 2-3 times higher than two other U.S. studies that measured young children [23,24]. One study included samples of 1.5-4 years old from all over the U.S., 2006-2007 [23], and one study focused on 12-36 month toddlers in North Carolina, 2009-2010 [24]. The PBDE serum levels obtained in those two studies were similar to each other with the exception of BDE-153.

Results are mixed when comparing concentrations in adult serum. Our concentrations are very similar to the U.S. average levels and actually lower than those for individuals from California collected in the National Health and Nutrition Examination Survey (NHANES) 2003-2004 [5]. Two potential reasons that our concentrations may be lower than California values measured by NHANES are that our population had a relatively high socioeconomic status, and most PBDE congeners’ serum levels have been found to be lower in individuals with a higher socioeconomic status, except for BDE-153 [25,26]. Second, pentaBDE concentrations may be decreasing over time based on the phasing out of pentaBDEs in furniture sold beginning in 2005 [27]. The concentrations measured in this study are significantly higher than those measured for adult, pregnant farm-workers in California; however, a significant fraction of the latter group was born outside of the U.S. and those born outside of the U.S. have been reported to have lower serum levels of PBDEs [7,8,23]. In addition, except for BDE-209, the older adults’ serum levels measured in our study were always slightly higher than the parents of young children, though not statistically significant. One assumption is that most of the parents of young children were female, who had recently been pregnant. Child birth and breastfeeding could reduce women’s body burden of PBDEs [28,29]. This fact combined with the phase-out of pentaBDE and octaBDE in 2005 indicates that the young mothers’ body burden may not return to the same level as before their pregnancy.

Because blood is difficult to obtain from children and expensive to analyze, we hypothesized that family members who share the same indoor environment and have a similar diet may have correlated PBDE serum concentrations. Based on our findings, serum PBDE concentrations from a parent may be a good indicator of his/her child’s levels, which could reasonably be used for a classification of children into low/medium/high categories in an epidemiology study. We note that determining the amount of time each family member spends at home and the commonality of their diets likely influences the level of correlation between family members. Similarly, results from older couples suggest that the serum concentrations of one person could be indicative of the exposure of other adults from the same household, however, for adults, occupational exposure should be considered.

We also report year-to-year correlations of PBDE serum concentrations. The results of the regression analysis indicate that, as expected, a one-time measurement could indicate the relative exposure level in cross-sectional profile. However, we saw a slight downward trend for the pentaBDEs, becoming significant for BDE-28 and 100. For the children, this may be due in part to the children growing, which has resulted in decreasing trends in previous studies [7,30]. Also, as PBDEs have been banned in U.S. and furniture with PBDEs continues to be removed from homes as it wears out and is replaced, the blood concentrations of PBDEs are expected to keep decreasing gradually. Meanwhile, along with the decreasing use of PBDEs, the chemicals used as flame retardants to replace PBDEs keep changing, which requires further attention [31].

Numerous studies have found correlations between PBDE serum concentrations and house dust concentrations [32-35]. Lorber [9] reviewed literature and concluded that residential dust contributed 82% of the estimated intake of PBDEs, while food intake is minor. Our study confirmed the predominant contribution of indoor sources as measured by a floor wipe for some congeners. However, serum concentrations of BDE-153 were not associated with floor wipe concentrations.

Dietary ingestion also contributes to PBDE exposure. We found the intake frequency of a few types of food significantly contributed to serum concentration of PBDEs and improved the power of predicting PBDE serum concentration. Fish, particularly salmon, has been reported to have the highest concentrations of PBDE residues in multiple market basket surveys [10,36,37]. Correlations were found between PBDE serum concentrations and fish consumption in a study of Norwegians, a population of high seafood consumers [38]. We found a relationship with tuna and white fish but not salmon. The fish consumption rate reported in our study was relatively low, with only 5% of our adult participants reporting that they eat salmon more than once per week. The consumption rates of other types of fish were similar.

Previous studies reported that, among the various food types, meat accounts for the highest body burden of PBDEs in the U.S. due to its high consumption rate [7,33,39,40]. On a per lipid basis, poultry and processed meats are found to have high PBDE concentrations [10]. Our study found a relationship with canned meat and meat entrées, which are considered processed meats, but not with beef or poultry. However, we found a negative association between pork intake and PBDE serum concentration. By looking at the scatter plot (not shown), the negative association is driven by a few households with high PBDE serum/low pork intake and a few households with low PBDE serum/high pork intake. PBDE levels in pork products vary greatly, and we did not differentiate pork products in our study. The lack of correlation with poultry is somewhat surprising as chicken fat intake was correlated with PBDE serum concentrations in a large, representative U.S. sample [39] and chicken intake was correlated with in another population of California children [7]. We note that our study did not ask portion size or differentiate by fat content. Given the complicity of different kinds of food products and different type of food processing/cooking styles, it is very difficult to characterize PBDE exposure by information obtained from a diet survey.

The model with housing characteristics found home value, an indicator of socioeconomic status, significant for the pentaBDEs. This result is consistent with other studies, even though our population has a higher socioeconomic status than the general population. The results are less clear for BDE-153, with one housing indicator, living in a single family home rather than an apartment pointing to higher socioeconomic groups having higher levels, and one indicator, rent/own, pointing the other way. Previous studies have found higher levels of BDE-153 in higher socioeconomic populations [24]. The age and proportion of carpeted floor, and presence of carpet padding may also influence PBDE levels but were not quantified in this study. The housing characteristics we examined cannot replace a measured environmental concentration in predicting PBDE serum concentrations.

The results of this study are subject to several limitations. First, the participants of this study had a relatively higher education level than the general population, and thus may not represent the general population of California. Second, the sample sizes for the correlation analysis among family members and temporal correlation were small and need further validation. Third, even though we identified a number of significant predictors of PBDE serum concentrations, the percent of total prediction error was explained by the model is somewhat low. One possible reason is that we used food intake frequency without portion size or details on fat content, so the estimation may not be precise enough to show the association. Also, additional important factors exist, which might be related to other sources, human activities, or physiologic factors such as BMI that could influence the absorption, distribution, mechanism and elimination of PBDEs from human body.

## Conclusions

This study contributes information on PBDE serum concentrations in different age classes, especially two sensitive groups, young children and seniors. Based on our analyses, age class, residential dust, home value, and intake frequency of canned meat, meat entrée, and tuna and white fish, were significant predictors for serum concentrations of individual PBDE congeners. Children had significantly higher serum levels of pentaBDEs than adults. Floor wipe concentrations were significantly associated with serum levels of BDE-47, 99, 100, 154, and 209, but not for BDE-153. Also, for the first time, we found a generally high correlation between the PBDE serum concentrations of the family members living in the same household (parent vs. child, and between spouses), which may be useful in modeling PBDE exposure. Decreasing temporal trends in PBDEs should be considered when characterizing long-term exposures.

### Supporting information available

Details on limit of detection; demographics of study population; and additional results of predictor models are available free of charge at http://pubs.acs.org.

## Additional files

Additional file 1: Table S1. Limit of Detection (LOD) of BDE congeners in serum samples. Additional file 2: Table S2. Demographics of study population. Additional file 3: Table S3. Spearman correlation coefficients among serum concentrations of major BDE congeners (N=217). Additional file 4: Table S4. Results of multiple regression model predicting BDE serum concentration with the log-transformed BDE concentration in the floor wipes. Additional file 5: Table S5. Results of multiple regression model predicting log-transformed serum concentration of BDEs with housing characteristics.

## Abbreviations

PBDE: Polybrominated diphenyl ethers
SUPERB: Study of Use of Products and Exposure- Related Behavior
CDC: Centers for Disease Control and Prevention
US EPA: United States Environmental Protection Agency
LOD: Limit of detection
GM: Geometric means
MLE: Maximum likelihood estimation
NHANES: National Health and Nutrition Examination Survey
