# Supplementary material for: Polybrominated diphenyl ether serum concentrations in a Californian population of children, their parents, and older adults: an exposure assessment study
Source: Environ Health. 2015 Mar 14;14:23. doi: 10.1186/s12940-015-0002-2 (PMC4381357; doi:10.1186/s12940-015-0002-2)
Supplement: Additional file 1: Table S1. — Limit of Detection (LOD) of BDE congeners in serum samples. [file 12940_2015_2_MOESM1_ESM.docx]

Limit of Detection (LOD) of BDE congeners in serum samples.

| Congeners | N | Arithmetic Mean | Std Dev | 10th% | 90th% | Range |
| --- | --- | --- | --- | --- | --- | --- |
| BDE17 | 279 | 0.51 | 0.28 | 0.30 | 0.60 | 0.2~3.8 |
| BDE28 | 279 | 0.51 | 0.28 | 0.30 | 0.60 | 0.2~3.8 |
| BDE47 | 279 | 0.83 | 0.46 | 0.50 | 1.10 | 0.4~6.1 |
| BDE66 | 279 | 0.51 | 0.28 | 0.30 | 0.60 | 0.2~3.8 |
| BDE85 | 279 | 0.51 | 0.28 | 0.30 | 0.60 | 0.2~3.8 |
| BDE99 | 279 | 0.58 | 0.32 | 0.40 | 0.70 | 0.3~4.3 |
| BDE100 | 279 | 0.51 | 0.28 | 0.30 | 0.60 | 0.2~3.8 |
| BDE154 | 279 | 0.51 | 0.28 | 0.30 | 0.60 | 0.2~3.8 |
| BDE153 | 279 | 0.51 | 0.28 | 0.30 | 0.60 | 0.2~3.8 |
| BDE183 | 279 | 0.51 | 0.28 | 0.30 | 0.60 | 0.2~3.8 |
| BDE209 | 273 | 5.01 | 2.63 | 3.30 | 6.40 | 2.3~37.6 |
