# Supplementary material for: Polybrominated diphenyl ether serum concentrations in a Californian population of children, their parents, and older adults: an exposure assessment study
Source: Environ Health. 2015 Mar 14;14:23. doi: 10.1186/s12940-015-0002-2 (PMC4381357; doi:10.1186/s12940-015-0002-2)
Supplement: Additional file 2: Table S2. — Demographics of study population. [file 12940_2015_2_MOESM2_ESM.docx]

Demographics of study population*

| Demographics | Northern California  Family Cohort^a^ | Central California  Older Adult Cohort |
| --- | --- | --- |
| N of Households | 90 | 49 |
| **Adult Sex**- female | 81 (90%) | 34 (69%) |
| **Adult Age** (Median: year) | 38.5 | 67.3 |
| **Child Sex-** female | 45 (50%) | — |
| **Child Age** (Median: year) | 5.5 | — |
| **Education** |  |  |
| High school | 8 (9%) | 5 (10%) |
| College degree or some college | 58 (64%) | 39 (80%) |
| Master, Doctor, and professional degree | 23 (26%) | 5 (10%) |
| **Job Status** |  |  |
| Employed | 38 (42%) | 18 (37%) |
| Unemployed (including stay-at-home parents) | 44 (49%) | 30 (61%) |
| Other | 6 (7%) | 1 (2%) |
| **Race/Ethnicity** |  |  |
| White (not Hispanic) | 60 (67%) | 37 (76%) |
| Asian (not Hispanic) | 7 (8%) | 0 (0%) |
| Black (not Hispanic) | 2 (2%) | 1 (2%) |
| Other (not Hispanic) | 7 (8%) | 5 (10%) |
| Hispanic (all races) | 13 (14%) | 6 (12%) |
| **Foreign Born** | 20 (22%) | 3 (6%) |
| **Homeowner** | 71 (79%) | 44 (90%) |
| **House Type** |  |  |
| Single house detached | 75 (83%) | 37 (76%) |
| Single attached house | 3 (3%) | 9 (18%) |
| Apartment | 11 (12%) | 3 (6%) |
| **Neighborhood Type** |  |  |
| Commercial | 0 (0%) | 1 (2%) |
| Residential | 72 (80%) | 41 (84%) |
| Rural | 5 (6%) | 2 (4%) |
| Combination of above | 11 (12%) | 5 (10%) |
| **Number of Children in the Household** (median) | 2 | 0 |

^a^Demographic information was missing for one household.
