# Supplementary material for: Polybrominated diphenyl ether serum concentrations in a Californian population of children, their parents, and older adults: an exposure assessment study
Source: Environ Health. 2015 Mar 14;14:23. doi: 10.1186/s12940-015-0002-2 (PMC4381357; doi:10.1186/s12940-015-0002-2)
Supplement: Additional file 3: Table S3. — Spearman correlation coefficients among serum concentrations of major BDE congeners (N=217). [file 12940_2015_2_MOESM3_ESM.docx]

Spearman correlation coefficients among serum concentrations of major BDE congeners (N=217)

|  | BB-153 | BDE-28 | BDE-47 | BDE-99 | BDE-100 |
| --- | --- | --- | --- | --- | --- |
| BDE-28 | -0.09 |  |  |  |  |
| BDE-47 | -0.16^*^ | 0.85^**^ |  |  |  |
| BDE-99 | -0.19^**^ | 0.78^**^ | 0.96^**^ |  |  |
| BDE-100 | -0.17^*^ | 0.77^**^ | 0.95^**^ | 0.94^**^ |  |
| BDE-153 | <0.01 | 0.53^**^ | 0.63^**^ | 0.64^**^ | 0.75^**^ |

^*^ *p*<0.05; ^**^*p* <0.01.
