# Supplementary material for: Polybrominated diphenyl ether serum concentrations in a Californian population of children, their parents, and older adults: an exposure assessment study
Source: Environ Health. 2015 Mar 14;14:23. doi: 10.1186/s12940-015-0002-2 (PMC4381357; doi:10.1186/s12940-015-0002-2)
Supplement: Additional file 4: Table S4. — Results of multiple regression model predicting BDE serum concentration with the log-transformed BDE concentration in the floor wipes. [file 12940_2015_2_MOESM4_ESM.docx]

Results of multiple regression model predicting BDE serum concentration with the log-transformed BDE concentration in the floor wipes^a^

|  | BDE-47  (N=187) | | BDE-99  (N=184) | | BDE-100 (N=185) | | BDE-153  (N=187) | | BDE-154  (N=187) | | BDE-209  (N=183) | |
| --- | --- | --- | --- | --- | --- | --- | --- | --- | --- | --- | --- | --- |
| Effect | Estimate (SE) | *p* value | Estimate (SE) | *p* value | Estimate (SE) | *p* value | Estimate (SE) | *p* value | Estimate (SE) | *p* value | Estimate (SE) | *p* value |
| Intercept | 2.71(0.12) | <.01 | 1.05(0.13) | <.01 | 1.07(0.13) | <.01 | 1.95(0.13) | <.01 | -1.13(0.31) | <.01 | -2.56(0.44) | <.01 |
| Age class (reference=parents of young children) | | | |  |  |  |  |  |  |  |  |  |
| Children | 1.14(0.09) | <.01 | 1.29(0.11) | <.01 | 1.18(0.10) | <.01 | 0.89(0.10) | <.01 | 1.73(0.40) | <.01 | 1.25(0.53) | 0.02 |
| Older adults | 0.27(0.17) | 0.12 | 0.10(0.19) | 0.59 | 0.19(0.18) | 0.30 | 0.09(0.21) | 0.66 | 0.37(0.39) | 0.34 | 1.37(0.55) | 0.01 |
| Floor wipe concentration (pg/cm^2^) | 0.03(0.01) | <.01 | 0.04(0.01) | <.01 | 0.15(0.04) | <.01 | -0.01(0.02) | 0.55 | 0.82(0.27) | <.01 | 0.003(0.003) | 0.20 |
| R-square | 0.30 |  | 0.28 |  | 0.28 |  | 0.10 |  | 0.25 |  | 0.09 |  |

Note: BDE-47, 99, 100, 153 were analyzed by generalized linear mixed-effect model, and BDE-154 and 209 were analyzed by logistic regression model due to their low detection. R-square of the generalized linear mixed-effect model was calculated by comparing with model with intercept only; and R-square of the logistic regression model was the max-scaled R-square.
