# Supplementary material for: Polybrominated diphenyl ether serum concentrations in a Californian population of children, their parents, and older adults: an exposure assessment study
Source: Environ Health. 2015 Mar 14;14:23. doi: 10.1186/s12940-015-0002-2 (PMC4381357; doi:10.1186/s12940-015-0002-2)
Supplement: Additional file 5: Table S5. — Results of multiple regression model predicting log-transformed serum concentration of BDEs with housing characteristics. [file 12940_2015_2_MOESM5_ESM.docx]

Results of multiple regression model predicting log-tranformed serum concentration of BDEs with housing characteristics^a^

| Factor | BDE47 | | BDE99 | | BDE100 | | BDE153 | |
| --- | --- | --- | --- | --- | --- | --- | --- | --- |
|  | Estimate (STDerr) | Pr > \|t\| | Estimate (STDerr) | Pr > \|t\| | Estimate (STDerr) | Pr > \|t\| | Estimate (STDerr) | Pr > \|t\| |
| Intercept | 3.21(0.28) | <0.01 | 1.50(0.31) | <0.01 | 1.48(0.29) | <0.01 | 1.61(0.31) | <0.01 |
| Age class (ref = parents of young children) | | | | | | | | |
| Young children | 1.13(0.08) | **<0.01** | 1.27(0.10) | **<0.01** | 1.20(0.09) | **<0.01** | 0.86(0.10) | **<0.01** |
| Older adults | -0.10(0.20) | 0.62 | -0.24(0.22) | 0.28 | -0.11(0.21) | 0.59 | -0.01(0.23) | 0.95 |
| Apartment (vs. SFH) | -0.30(0.25) | 0.23 | -0.24(0.27) | 0.39 | -0.39(0.26) | 0.14 | -0.62(0.28) | **0.03** |
| Home built after 1977 | 0.16(0.17) | 0.37 | 0.15(0.19) | 0.44 | 0.15(0.18) | 0.41 | -0.04(0.20) | 0.86 |
| Home rent (vs. owned) | 0.32(0.22) | 0.15 | 0.27(0.24) | 0.28 | 0.38(0.23) | 0.11 | 0.51(0.25) | **0.05** |
| House value ($1,000,000) | -0.68(0.29) | **0.02** | -0.64(0.32) | **0.05** | -0.63(0.30) | **0.04** | -0.34(0.32) | 0.29 |
| Home size (1000ft^2^) | 0.05(0.13) | 0.71 | 0.05(0.14) | 0.73 | 0.08(0.13) | 0.53 | 0.22(0.15) | 0.13 |
| # of upholster furniture between 1980 and 2004 in the sampled room | 0.07(0.06) | 0.20 | 0.07(0.06) | 0.26 | 0.04(0.06) | 0.49 | 0.05(0.06) | 0.40 |

|  | BDE154 | | BDE209 | |
| --- | --- | --- | --- | --- |
| Factor | Estimate (95% CIs) | Pr > ChiSq | Estimate (95% Cis) | Pr > ChiSq |
| Intercept | 0.57(N/A) | 0.30 | 0.12(N/A) | <0.01 |
| Age effect (ref = parents of young children) | | | | |
| Young children | 4.70(2.32,9.54) | **<0.01** | 3.26(1.29,8.27) | **0.01** |
| Older adults | 0.99(0.45,2.16) | 0.97 | 2.66(0.90,7.82) | 0.08 |
| Apartment (vs. SFH) | 0.64(0.24,1.68) | 0.36 | 0.86(0.26,2.85) | 0.81 |
| Home built after 1977 | 1.17(0.61,2.23) | 0.64 | 1.49(0.67,3.28) | 0.32 |
| Home rent (vs. owned) | 1.83(0.79,4.24) | 0.16 | 1.57(0.58,4.26) | 0.37 |
| House value ($1,000,000) | 0.42(0.14,1.21) | 0.11 | 0.41(0.08,1.98) | 0.27 |
| Home size (1000ft^2^) | 1.20(0.75,1.90) | 0.45 | 1.15(0.64,2.04) | 0.64 |
| # of upholster furniture between 1980 and 2004 in the sampled room | 1.12(0.91,1.38) | 0.29 | 0.92(0.70,1.21) | 0.55 |

^a^ For BDE47 to 153, data were analyzed using generalized linear regression model; for BDE154 and 209, data were analyzed based on detectable or not, using multiple logistic regression model.
